# Physiological Changes in Subjects Exposed to Accidental Hypothermia: An Update

**DOI:** 10.3389/fmed.2022.824395

**Published:** 2022-02-23

**Authors:** Lars J. Bjertnæs, Torvind O. Næsheim, Eirik Reierth, Evgeny V. Suborov, Mikhail Y. Kirov, Konstantin M. Lebedinskii, Torkjel Tveita

**Affiliations:** ^1^Department of Clinical Medicine, Faculty of Health Sciences, Anesthesia and Critical Care Research Group, University of Tromsø, UiT The Arctic University of Norway, Tromsø, Norway; ^2^Division of Surgical Medicine and Intensive Care, University Hospital of North Norway, Tromsø, Norway; ^3^Department of Clinical Medicine, Faculty of Health Sciences, Cardiovascular Research Group, University of Tromsø, UiT The Arctic University of Norway, Tromsø, Norway; ^4^Science and Health Library, University of Tromsø, UiT The Arctic University of Norway, Tromsø, Norway; ^5^The Nikiforov Russian Center of Emergency and Radiation Medicine, St. Petersburg, Russia; ^6^Department of Anesthesiology and Intensive Care, Northern State Medical University, Arkhangelsk, Russia; ^7^Department of Anesthesiology and Intensive Care, North-Western State Medical University named after I.I. Mechnikov, St. Petersburg, Russia; ^8^Federal Research and Clinical Center of Intensive Care Medicine and Rehabilitology, Moscow, Russia

**Keywords:** accidental hypothermia, cardiopulmonary resuscitation, hypothermic cardiac arrest, temperature regulation, extracorporeal life support, hibernating animals, oxygen-saving mechanisms, systemic inflammatory response syndrome

## Abstract

**Background:**

Accidental hypothermia (AH) is an unintended decrease in body core temperature (BCT) to below 35°C. We present an update on physiological/pathophysiological changes associated with AH and rewarming from hypothermic cardiac arrest (HCA).

**Temperature Regulation and Metabolism:**

Triggered by falling skin temperature, Thyrotropin-Releasing Hormone (TRH) from hypothalamus induces release of Thyroid-Stimulating Hormone (TSH) and Prolactin from pituitary gland anterior lobe that stimulate thyroid generation of triiodothyronine and thyroxine (T4). The latter act together with noradrenaline to induce heat production by binding to adrenergic β3-receptors in fat cells. Exposed to cold, noradrenaline prompts degradation of triglycerides from brown adipose tissue (BAT) into free fatty acids that uncouple metabolism to heat production, rather than generating adenosine triphosphate. If BAT is lacking, AH occurs more readily.

**Cardiac Output:**

Assuming a 7% drop in metabolism per °C, a BCT decrease of 10°C can reduce metabolism by 70% paralleled by a corresponding decline in CO. Consequently, it is possible to maintain adequate oxygen delivery provided correctly performed cardiopulmonary resuscitation (CPR), which might result in approximately 30% of CO generated at normal BCT.

**Liver and Coagulation:**

AH promotes coagulation disturbances following trauma and acidosis by reducing coagulation and platelet functions. Mean prothrombin and partial thromboplastin times might increase by 40–60% in moderate hypothermia. Rewarming might release tissue factor from damaged tissues, that triggers disseminated intravascular coagulation. Hypothermia might inhibit platelet aggregation and coagulation.

**Kidneys:**

Renal blood flow decreases due to vasoconstriction of afferent arterioles, electrolyte and fluid disturbances and increasing blood viscosity. Severely deranged renal function occurs particularly in the presence of rhabdomyolysis induced by severe AH combined with trauma.

**Conclusion:**

Metabolism drops 7% per °C fall in BCT, reducing CO correspondingly. Therefore, it is possible to maintain adequate oxygen delivery after 10°C drop in BCT provided correctly performed CPR. Hypothermia may facilitate rhabdomyolysis in traumatized patients. Victims suspected of HCA should be rewarmed before being pronounced dead. Rewarming avalanche victims of HCA with serum potassium > 12 mmol/L and a burial time >30 min with no air pocket, most probably be futile.

## Introduction

Accidental hypothermia (AH) is a fall in body core temperature (BCT) to below 35°C after exposure to cold or decrease in metabolic rate (1, 2). Volunteers subjected to hypothermia may present with reduced consciousness when approaching a BCT of 33.5°C. Simultaneously, the electroencephalogram (EEG) shifts to less alpha activity and more theta and beta frequencies. Most people lose consciousness at a BCT of 30°C and EEG mostly appears isoelectric below 20°C (3, 4).

Humans react to mild hypothermia (BCT of 35–32°C) with tachypnea, peripheral vasoconstriction and increased tendency of atrial fibrillation. Arrhythmia risk increases as the temperature drops further. Severe hypothermia (below 28°C) is associated with falling respiratory rate, increased tidal volumes, reduced oxygen consumption and increased risks of more severe cardiac dysrhythmias (atrioventricular block, ventricular fibrillation, asystole, or pulseless electrical activity). Estimated mortality rate after AH varies between 30 and 80%. In deep AH (below 20°C), nearly all patients present with asystole (5–7).

In a historical perspective, AH has contributed to the outcome of wars. While crossing the Alps during his siege of Italy (218–203 BC), Carthaginian general Hannibal lost an estimated half of his army of more than 100.000, and Napoleon when attacking Russia in 1812, left behind more than half a million men in battle, or because of malnourishment and hypothermia combined. Nearly one million German soldiers perished during the battle of Stalingrad in World War II, and the subsequent retreat in February 1943 (8, 9).

The lowest BCT noticed in survivors of HCA, is 11.8°C in a 2$\frac{1}{2}$ year old boy and 13.7°C in a 29 year old female skier, respectively (10, 11). However, the temperature limit, to which the human body can be actively cooled, followed by hours of hypothermic cardiac arrest (HCA), and still with a maintained potential of successful resuscitation, is unknown. Hypothermia with BCT of 9 and 4.2°C, followed by 1 hour of cardiac arrest (CA), was induced as adjunct therapy in two patients suffering from cancer. Although HCA had no beneficial effects on the malignancies, the patients underwent successful resuscitations with no sequela due to the cooling *per se* (12, 13).

Prognosis of hypothermic cardiac arrest (HCA) depends on the circumstances causing AH, the quality and length of cardiopulmonary resuscitation (CPR, and the types of treatments given from the scene of accident to a tertiary hospital providing rewarming by means of extracorporeal life support (ECLS) (14, 15). However, if such a hospital could not be reached, survival from HCA has been reported even after 6$\frac{1}{2}$ h of manually performed cardiopulmonary resuscitation (CPR) at a local hospital (16). Notably, survival rate differs between victims of witnessed CA being able to breathe while gradually loosing body temperature, and those developing asphyxia and acidosis before the heart stops (17–19). Particularly high mortality rates were reported in traumatized patients with a BCT below 32°C, in whom mortality reached nearly 100% (20).

As activities in circumpolar areas increase, AH affects not only the poor and the elderly, but also tourists, hikers, skiers, mountain climbers and workers exploiting natural resources. Often taking place on remote locations, these activities necessitate that health personnel have a good knowledge of the physiology and pathophysiology of AH and update themselves about logistics and treatment algorithms and train together as teams (21). Our aim is to update the readers on physiological changes occurring in various organ systems, as a knowledge basis for rewarming victims of AH and HCA. Although the topic recently was reviewed by one of us, the different emphasis should make this paper be considered as complementary (22).

## Background and Methods

In April 2016, we started a collaboration in studying the management of accidental hypothermia between Anesthesia and Critical Care Research Group, Department of Clinical Medicine, Faculty of Heath Sciences, The Arctic University of Norway, Tromsø, Norway and The Department of Anesthesiology and Intensive Care Medicine, North-Western State Medical University named after I.I. Mechnikov, St. Petersburg and Department of Anesthesiology and Intensive Care, Northern State Medical University, Arkhangelsk, Russia. We agreed on starting the collaboration with publishing our local Norwegian guidelines for rewarming victims of HCA in Anesteziologia and Reanimatologia, the official Journal of the Russian Society of Anesthesiologists (23). Moreover, we aimed at authoring a review and meta-analysis on rewarming victims of HCA by means of extracorporeal life support (ECLS) (24), and the present update on the physiology and pathophysiology of accidental hypothermia and HCA. The latter two projects are registered in PROSPERO international prospective register of systematic reviews (registration no. 47,934). These projects spawned a systematic search for references across two databases (Medline and EMBASE), resulting in close to 1,600 references of interest, made available for all co-authors by use of Endnote (Endnote TM 97.4; Thompson Reuters. Toronto, ON, Canada). In addition, we performed a directed and goal-oriented search for additional references in the same databases, using key scientific phrases from each of the headline partitioning in this update. References were screened before making a discretionary consensus-based selection of which of them we found relevant to include in each individual paper.

The present update originates from our collective experiences from clinical practice and experimental activities. Six of the authors have a background as consultants in anesthesia with cardiothoracic anesthesia and intensive care medicine, including extracorporeal membrane oxygenation (ECMO), as main fields of interest. Two of the authors, additionally have worked as consultants in the governmental Norwegian air ambulance system (LB and TN). All the authors have doctoral degrees or have spent several years in experimental physiology and/or pathophysiology research.

The following key search phrases were used in combination with hypothermia, in the additional directed and goal-oriented search for additional references: temperature regulation, myocardial function^*^, drug^*^, CPR, cardiopulmonary resuscitation, respiration, respiratory, breath holding, acidosis, coagulation, fibrinolysis, kidney, liver function. For complete information on search methodology [see (24)].

## Hypothermia-Induced Changes in Organ Functions

### Temperature Regulation and Metabolism

Temperature homeostasis is a thoroughly regulated balance between heat production and dissipation. AH occurs when heat loss is greater than heat production. The drop in BCT either results from decreased metabolic rate or exposure to cold without capability of increasing heat production sufficiently, giving rise to “chronic hypothermia” (2). Patients abusing drugs and alcohol or suffering from cancer or sepsis are often disposed to malabsorption and endangered by AH due to decreased thermogenesis. In some countries, particularly the elderly are prone to AH because of poor living quarters, malnourishment, neglected illnesses and lack of physical activity (25, 26).

Skeletal muscles generate heat under physical activity and by shivering thermogenesis, which might increase heat production by up to five times. An integrated response to cold triggers formation of Thyrotropin-Releasing Hormone (TRH) in the preoptic area of hypothalamus. Acting *via* secretion of Thyroid stimulating hormone (TSH), TRH promotes synthesis and release of triiodothyronine (T_3_) and its prohormone, thyroxine (T_4_) in the thyroid gland. These hormones work in concert with increasing noradrenergic stimulation to enhance heat production (27).

Neonates of seals that are born on the ice, generate heat under adrenergic stimulation of metabolism of brown adipose tissue (BAT) (28). This is localized in the regions of the back and shoulders (29). When exposing babies to cold, noradrenaline binds to adrenergic β_3_-receptors of fat cells that trigger degradation of triglycerides into free fatty acids (FFA). The latter interact with mitochondrial uncoupling protein-1 (UCP1), which uncouples metabolism to produce heat, rather than adenosine triphosphate (ATP). Therefore, if BAT is lacking, for instance, in premature babies, hypothermia occurs more easily (30, 31). In adults, metabolic rate normally rises by increasing metabolism in skeletal muscles (shivering thermogenesis), and in various other tissues as the liver and the intestines. However, the last few years, investigators have shown that BAT also plays an important role in heat generation in adults, as demonstrated by means of ^18^F - FDG-positron emission tomography/computed tomography (PET/CT) imaging (32, 33).

According to recent investigators, the amount of BAT can be linked to the body mass index (BMI) and the season of conception. By transferring these ideas to an experimental model on mice, the authors showed that cold exposure of males before mating resulted in increased systemic metabolism and protection of male offspring from diet-induced obesity (34). The investigators suggested that the improved metabolic condition of the offspring was due to enhanced BAT formation, increased neurogenesis, and noradrenaline release into BAT during cold exposure. They concluded that epigenetic changes might occur that can even improve cold-adaption of future generations in sperms of individuals exposed to cold climate (34).

Fur animals reduce heat dissipation by means of piloerection, which widens the air layer insulating the skin. This α_1_-adrenergic and phylogenetically old heat conservation mechanism also occurs in humans, although being of little practical significance (35).

Researchers have suggested to increase body temperature of victims of immersion or exposure to cold by supplying them with a pill that can increase heat production, such as 3,4-methylenedioxymethamphetamine (MDMA, “Ecstasy”) (36). Clinical experiments indicate that the MDMA-induced elevation of human body temperature partially depend on release of norepinephrine, which involves enhanced metabolic heat generation concomitant with cutaneous vasoconstriction, resulting in impaired heat dissipation. However, use of this recreational drug in an emergency should be strongly discouraged.

### Can We Learn From Hibernating Animals?

Mammals, such as hedgehogs, bats, hamsters, squirrels and bears hibernate to save energy. The ground squirrel, for instance, hibernates through seven months of freezing temperatures, during which its heart rate and body temperature drops to approximately one beat per minute and nearly −3°C below the freezing point of water, respectively, while brain temperature remains above +0.7°C. During hibernation, circadian clock function is inhibited for periods of up to 13 days (37). Why blood does not freeze when undercooled remains a puzzle. Some experts believe that ground squirrels are able to remove nucleators from circulation that generate ice crystals in blood (38). However, although the mechanism behind this super cooler remains grossly unclear (39), it could be of potential use both for preservation of donated organs for transplantations and for provoking a state of “suspended animation” in severely traumatized patients while awaiting blood transfusion or organ transplantation (40, 41).

### Myocardial Changes During Hypothermia and After Rewarming

Hypothermia is frequently associated with myocardial dysfunction. Previous researchers noticed that when dogs were cooled to a BCT of 21°C, cardiac output (CO) decreased exponentially to 20% of its value at normal BCT and recovered to significantly below its control value upon rewarming whereas myocardial blood flow (MBF) returned to pre-hypothermic values (42). Tveita et al. reported a 37% decrease in CO upon cooling of dogs to 25°C, but in their experiments neither CO nor MBF returned to their pre-hypothermic control values upon rewarming (43). By lowering the aortic arch temperature in rats to 13–15°C, Tveita et al. observed that CO fell to 13% of its control value. Assuming a 7% drop in metabolism per °C fall in BCT a spontaneously breathing patient should experience approximately a 75% reduction of metabolism following a fall in BCT to 26°C and a corresponding decrease in CO (44). Consequently, if cardiac arrest occurs at or below this temperature, it is possible to maintain adequate oxygen delivery by means of external CPR. In dogs, correctly performed heart-lung resuscitation resulted in 42% of the CO measured with Fick's principle prior to ventricular fibrillation (45). In patients with cardiac arrest, Fodden and coworkers obtained a median cardiac index (CI) of 3.2 L min^−1.^m^−2^ as assessed by Doppler aortovelography during external cardiac compression performed by experienced personnel as opposed to 1.2 L min^−1^ m^−2^ when CPR was carried out by unexperienced operators (46). The electrocardiogram (ECG) changes characteristically during hypothermia. Patients may present with sinus bradycardia, AV-block, widened QRS complex, QT prolongation or pulseless electrical activity (PEA) (47, 48). Higuchi and co-workers analyzed the prevalence of so-called Osborne J waves (hypothermia-associated notches on the QRS complex) in 60 AH patients. In 50% of the patients, the investigators found Osborne J waves that disappeared upon rewarming to pre-hypothermic BCT. The J waves tended to occur with higher frequency and amplitude in patients with the lowest BCT. However, the authors found no associations between J waves and fatal arrhythmias (49).

In 2012, Shattock and Tipton introduced the concept “autonomic conflict,” which is a potential mechanism of arrhythmias that frequently causes unexpected deaths in cold water due to simultaneous powerful stimulation of both the sympathetic and the parasympathetic nervous systems (50, 51). According to the authors, “autonomic conflict” occurs suddenly upon rapid submersion and breath holding in water at temperatures <15°C. This activates two powerful autonomic responses, the cold shock response, and the diving response. The former consists of reflexes driven by cutaneous cold-receptors and is characterized by sympathetically mediated tachycardia, a respiratory gasp, uncontrollable hyperventilation, peripheral vasoconstriction, and hypertension. The latter reflex triggers a strong excitation of cardiac vagal motor neurons *via* M_2_ acetylcholine receptors causing sinus bradycardia and expiratory apnea. Reflex inhibition of central respiratory neurons and excitation of sympathetic vasoconstrictor neurons make up the complete picture. These responses generate vasoconstriction mainly in the trunk and the limbs, thereby prioritizing cerebral blood flow and oxygenation (50).

Despite the fact that cardiac contractility is partially restored after rewarming, cardiac function may be a limiting factor for survival after AH (52). The reduced contractility in deep hypothermia (BCT <15°C) is believed to be caused by reduced myocyte calcium (Ca^2+^) - sensitivity in association with increased phosphorylation of Troponin I (52–54). According to Kondratiev et al. alterations in Ca^2+^-handling resulted in Ca^2+^ - overload during hypothermia/rewarming, which may contribute to myocardial failure during and after rewarming (55). In contrast, in mild hypothermia in pigs (33°C), left ventricular contractility increases, as compared to the situation at 37°C, and diastolic relaxation appeared to be delayed independent of heart rate (56). However, another porcine study failed to show increased contractility, as determined by echocardiography and preload recruitable stroke work relationship. By contrast, in that investigation the heart showed increased duration of systole on the account of reduced ventricular filling and shortening of the diastole (57). Thus, apparently the increased contractility in mild hypothermia does not depend on increased Ca^2+^-transients in myocytes, but rather on increased myofilament responsiveness to calcium (58).

### Inotropic, Vasoactive and Antiarrhythmic Drugs in Hypothermic Subjects

Evidence is sparse concerning the effects of vasoactive-, antiarrhythmic- and inotropic drugs in victims of AH. Therefore, most recommendations are derived from animal experiments (59). After rewarming from deep AH, an acute cardiac failure, called “rewarming shock” threatens the patients. This is a progressive reduction of cardiac output (CO) in association with a decrease in arterial blood pressure. Inotropic drugs have been tested in attempts on preventing rewarming shock (60). However, the effect of anti-arrhythmic and inotropic medicines in victims of HCA seems ambiguous. Therefore, international guidelines are reluctant to recommend their use (61, 62).

Clinical experience indicates that the hypothermic heart is relatively unresponsive to defibrillation, pacing and vasoactive – and anti-arrhythmic drugs below a BCT of 30°C (62). Tveita and Sieck report from experiments on rats that adrenalin, given during normothermia, in doses that increased CO without affecting vascular resistance, gave rise to vasoconstriction, but failed to elevate a low CO when injected on hypothermic animals (60). In contrast, a recent meta-analysis of investigations aimed at studying return of spontaneous circulation (ROSC) in severely hypothermic animals with ventricular fibrillation, revealed that success rates were higher with application of vasopressor medications (i.e., adrenaline or vasopressin), as compared with placebo (63).

In normothermic conditions, adrenaline affects cardiac contractility by stimulating myocyte sarcolemmal β-adrenoceptors *via* cyclic adenosine monophosphate (cAMP) and protein kinase A pathways. This allows for a greater trans-sarcolemmal Ca^2+^ influx with each depolarization, which is partly responsible for the positive inotropic effect of adrenaline during normothermia. Relaxation is caused by re-uptake of Ca^2+^ into the sarcoplasmic reticulum by means of sarcolemmal ATPase and Na^+^/Ca^2+^ exchange (64). In pigs exposed to dopamine infusion during surface cooling to a BCT of 25°C, left ventricle stroke volume fell and systemic vascular resistance and heart rate increased concomitant with a four-fold rise in dopamine plasma concentration, as compared with the condition after rewarming (65).

Concerning antiarrhythmic therapy, hypothermic dogs with ventricular fibrillation treated with either amiodarone or bretylium demonstrated no significant difference in survival rate as compared with placebo-treated controls (66). Interestingly, researchers studying HCA in rats reported that the combined calcium sensitizer and phosphodiesterase 3 (PDE3) inhibitor, levosimendan, exerted positive inotropic effects during hypothermia and rewarming from a core temperature of 15°C (67, 68). However, in spite of documentation of promising effects on rats, we found only an anecdotal report confirming similar effects of levosimendan on cardiac function in humans (69). Thus, according to recent studies, administration of adrenaline might be futile during resuscitation of individuals with BCT below 30°C. Indications for dopamine and levosimendan as inotropic and vasoactive support for resuscitation of patients with HCA need to be further elucidated in experimental and clinical studies (70).

### Continuity of CPR in Victims of HCA

Prehospital, resuscitation of hypothermic patients should be rejected only if the cause of CA is attributable to a lethal injury, fatal illness, prolonged asphyxia, or incompressible chest stiffness (61). If diagnosed without pulses after checking for 1 min, CPR of a hypothermic patient should start immediately, and preferentially continue with a mechanical chest-compression device to avoid interruption during transport (71, 72). The European Resuscitation Council recommend the same technique for chest compression and ventilation rates for patients of HCA as for normothermic victims of CA. Moreover, if ventricular fibrillation (VF) persists after three attempts of defibrillation, further attempts should be postponed until the patient is warmed to a BCT above 30°C. Adrenaline injections also should be avoided when BCT is below 30°C. When BCT raises above 30°C, adrenaline administration intervals of 6–10 min during normothermia should be doubled (61). In patients with a BCT of between 28 and 20°C, or with unknown BCT, CPR should go on continuously for periods of at least 5 min, alternating with periods of no longer than 5 min without CPR. In victims of HCA with a BCT below 20°C, CPR should be interrupted maximally for 10 min p (73).

### Oxygen–Saving Mechanisms

In victims of asphyxia, like drowning and avalanches, respiration might be hampered before the heart stops, due to aspiration of liquids into the airways, or compression of the chest hindering lung ventilation. On the other hand, according to forensic investigators, 10–15% of drowned victims have “dry” airways because laryngeal spasm has prevented water from intruding into the airways (74–76). More than 80 years ago, Irwin and Scholander suggested that in diving animals, breath-holding triggers cardiovascular mechanisms that are established for saving oxygen by preferentially redistributing circulation to the myocardium and the brain, the organs most in need of continuous oxygen supply (77, 78). A heart catheterization study in humans showed that intermittent periods of apnea and face immersion (AFI) more than halved cardiac output (CO) concomitant with a doubling of systemic vascular resistance during ergometer bicycling at constant workload (79). While bicycling at the same workload, one of the test subjects demonstrated a 85% reduction in average blood velocity (ABV) in a radial artery concomitant with a 67% increase in ABV in a vertebral artery during exposure to AFI, as determined with a Doppler ultrasound velocity meter (80). Correspondingly, Kjeld et al. noticed that middle cerebral artery ABV more than doubled during AFI when exercising at 100 Watt (81). In 1999, Lindholm et al. found a correlation between cardiovascular responses and oxyhemoglobin desaturation rate, indicating a causal relationship between intensity of the cardiovascular responses to apnea and O_2_ -conservation, as reflected by a less steep decline in SaO_2_, as determined by pulse oximetry during breath-holding (82). An increase in vertebral artery ABV also might be triggered by a rise in PaCO_2_ secondary to apnea. In a real drowning situation, the increase in vertebral artery blood flow could potentially increase the rate of brain cooling due to heat exchange in the lungs concomitantly reducing the cerebral metabolic oxygen demand, thus prompting a more successful outcome from drowning, as suggested by Golden and recently demonstrated in the seal by Blix and co-workers (83, 84).

The AFI-induced increase in systemic arterial pressure (79), which is also observed in diving ducks, differs from that experienced in seals in whom there is no such increase while cardiac output decreases to ~1/10 of its value during normal breathing (85). We cannot decide to what extent the diving response protects people exposed to drowning or burial by avalanche from brain anoxia. However, case reports of successful resuscitation after more than 40 min of HCA after submersion, make such contributions likely (50, 86–88).

### Respiratory Effects of Hypothermia

In victims of AH, respiratory rate and depth decrease with falling BCT. Evolving hypoventilation causes CO_2_ accumulation, which gives rise to hypoxia and respiratory acidosis. Mucociliary function of the respiratory epithelium and the cough reflexes are depressed, thus predisposing for secrete stagnation and pneumonia (89). Studies in sheep cooled on femoral veno-arterial bypass revealed that lung mechanics are affected during cooling and rewarming. During deep hypothermia lung compliance dropped transiently, but normalized upon rewarming to 24–30°C, and decreased again on further warming (90). A retrospective study of mechanically ventilated patients exposed to therapeutic hypothermia after cardiac arrest, showed significant decreases in PaCO_2_ and airway pressure and increased lung compliance, as compared with normothermic controls (91). In full term infants subjected to whole body cooling because of hypoxic ischemic encephalopathy, PaO_2_/FIO_2_ ratio increased in concert with a decrease in PaCO_2_, which was interpreted as a result of reduced oxygen consumption and CO_2_ production at unchanged ventilation and constant PEEP levels during hypothermia (92, 93).

### Hypothermia-Induced Changes in pH

A neutral solution is defined as one containing equal numbers of [H^+^] and [OH^−^] ions, not as a solution of a pH of 7. In any solution, pH changes with temperature since the dissociation of H_2_O into [H^+^] and [OH^−^]ions is an endothermic reaction. When the temperature decreases, the concentration of [H^+^] falls in parallel with a raising pH. However, the ratio between [H^+^] and [OH^−^], remains unchanged and acid/base neutrality is maintained (94). The ratio of protonated to non-protonated intracellular proteins depends on neutrality rather than pH, and therefore remains constant, independent of temperature changes. In hypothermic patients, correction of pH by adjusting an apparent respiratory alkalosis at normal BCT, might lead to disruption of the normal extracellular/intracellular pH-gradient. This might cause distortion of intracellular neutrality, and derangement of cellular function.

As the solubility of O_2_ and CO_2_ in water increases with decreasing temperature, gaseous partial pressures above the liquid level fall as the temperature drops. Hemoglobin's affinity for oxygen also increases when the temperature falls. This results in a discrepancy between gaseous partial pressures and the contents of gases in blood. Ashwood and co-workers demonstrated that the oxygen content of blood is constant over a wide range of temperatures although PaO_2_ varies (95). Temperature corrections of partial pressures of O_2_ and CO_2_ in hypothermic patients might therefore lead to interventions that will induce unphysiological blood gas concentrations.

Before measuring blood gases, the blood gas analyzer warms the blood sample to 37°C. If BCT of the patient is below 37°C, PaO_2_, PaCO_2_ and pH will change during heating of the sample to 37°C. Thus, the values will not reflect the actual levels in the cold patient. Correcting formulas, allow us to use the values determined at 37°C for correction of any blood gas abnormalities. This kind of correction is named the pH-stat-strategy. However, such corrections can be physiologically delusive. Alternatively, according to the so-called alpha-stat strategy, we accept the values measured at 37°C without any correction, When we use the pH-stat strategy, gaseous CO_2_ often must be added to the inspiration gas mixture to achieve normal PaCO_2_ and pH values, as compared with the α-stat strategy (96).

Carbon dioxide (CO_2_) is a cerebral vasodilator. Increased levels of CO_2_ have a potential of increasing cerebral perfusion beyond metabolic requirements by offsetting cerebral autoregulation (CAR). When using CPB, the increased cerebral blood flow is associated with an increased risk of cerebral emboli (97). Reduced pH and increased CO_2_-levels counteract a leftward shift of the oxyhemoglobin dissociation curve, facilitating release of O_2_ to the tissues. (98, 99). Gaasch and co-workers noticed impaired CAR during deep hypothermia in pigs. However, despite a decrease in MAP and CPP, brain oxygenation increased, most likely due to a decrease in brain metabolism (100).

Rats subjected to transient closure of the middle cerebral artery at normal BCT, subsequently followed by reperfusion for 5 h at 33°C, and treatment according to the pH-stat- strategy, had significantly better outcome as compared with animals exposed to an α-stat policy. These findings also were confirmed in piglets cooled to a brain temperature of 19°C and subsequently subjected to profound HCA for 90 min before being resuscitated on CPB (101, 102). In patients undergoing combined valve surgery with pH/PaCO_2_ - management following the pH-stat policy, investigators reported that CPB at BCT ≤ 32°C resulted in higher central venous oxygen saturation, but decreased cerebral tissue oxygenation, oxygen delivery and oxygen consumption, as compared with normothermic CPB (103). Abdul Aziz and Meduoye stratified outcome results from 16 best evidence reports. They suggested that the best management of acid-base balance in patients undergoing deep HCA during cardiac surgery depends upon the age of the patient. The authors concluded that the pH-stat protocol should be used in children and the alpha-stat strategy in adults (104).

### Blood Coagulation and Fibrinolysis

Victims of HCA often present with changes in the coagulation system. However, there is no consensus on whether these changes are due to asphyxia, acidosis, or hypothermia *per se*, or to a combination. Traumatized patients may suffer increased blood loss because of deranged coagulation. The mechanisms include activation of protein C, platelet dysfunction, fibrinogen depletion, and endothelial glycocalyx disruption. Hypothermia and acidosis that often accompany trauma, may amplify the coagulopathy. Researchers have demonstrated significant reductions in platelet function and coagulation system activity even after mild hypothermia (105, 106). However, evidence is conflicting and there is still a need to elucidate the effect on platelet function of hypothermia *per se*. Some investigators suggest that platelet aggregation even increases in mild hypothermia (107, 108). They believe that the increased bleeding rather results from coagulopathy, consistent with impaired thrombin generation (109).

In a cohort of 58 traumatized patients, the investigators found that half of them presented with life-threatening coagulopathy characterized by prothrombin time and partial thromboplastin time that were the double of those of the controls. Multiple logistic regression revealed four significant risk factors, (1) pH <7.10 with odds ratio (OR 12.3), (2) temperature <34°C (OR 8.7), (3) Injury Severity Score (ISS) >25 (OR 7.7) and (4) systolic blood pressure <70 mm Hg (OR 5.8). With all risk factors present, incidence of coagulopathy amounted to 98%. This indicates that in traumatized patients, even mild hypothermia may increase bleeding. Thus, protective measures against heat loss should have high priority already from the site of accident.

Tissue hypoxia can induce expression of tissue factor (TF) (110), thereby triggering the coagulation system. According to Østerud and Bjørklid, TF originates from TF-rich micro particles shedded from monocytes (111). Deep hypothermia followed by rewarming can lead to tissue factor (TF) release from damaged tissues, which triggers the formation of fulminant disseminated intravascular coagulation (DIC), as reported by Mahajan and co-workers (112). These investigators argue that hypothermia inhibits both platelet aggregation and coagulation in cases in which hypothermia occurs prior to asphyxia and cardiac arrest.

### Liver Function

In patients with HCA, liver function tests, such as serum aspartate aminotransferase (ASAT) and alanine aminotransferase (ALAT were used as markers for liver damage. Both increased significantly albeit without significant difference between survivors and non-survivors (113). The liver produces most of the coagulation factors. Hepatocytes synthesize fibrinogen, prothrombin, coagulation factors V, VII, IX, X, XI, XII, proteins C and S and antithrombin, and sinusoidal endothelial cells produce von Willebrand factor and factor VIII (114). Recently, researchers examined the effect of hypothermia on the coagulation cascade and found that mean prothrombin time and partial thromboplastin time increased significantly by ~40 and 60%, respectively. The authors conclude that hypothermia strongly inhibits the enzymatic reactions of the coagulation cascade even when coagulation factor levels were normal (115).

The liver also plays an important role in the metabolism and clearance of drugs from circulation, but displays reduced capability in patients undergoing therapeutic hypothermia (116). Let us, for instance, assess the metabolism of midazolam. In a study, eight patients were subjected to mild therapeutic hypothermia for 48 hrs, with seven normothermic controls (117). Each subject received midazolam 5 μg/kg/min intravenously. Normothermic subjects obtained steady state plasma concentration of ~1500 ng/mL. The hypothermic patients did not achieve steady state, but obtained a five-fold increase in midazolam plasma concentrations when BCT fell <35°C. Pharmacokinetic analysis showed a >100-fold decrease in systemic clearance of midazolam when BCT fell <35°C. Since midazolam degradation depends on CYP3A4 and CYP3A5 activities, the marked increase in midazolam concentration were due to depressed activity of the cytochrome P450 family of detoxification enzymes. These enzymes are responsible for the clearance of a great variety of commonly used drugs (118).

### Kidney Function, Electrolytes and Fluid Balance

By lowering BCT of rats by 10°C, renal blood flow (RBF) decreased by nearly 50% followed by an almost complete restoration of control values after rewarming (119). Systemic blood pressure remained unchanged throughout the experiment. The reduced RBF was due to a 75% increase in vascular resistance, due to constriction of afferent arterioles, combined with increased blood viscosity. A decrease in glomerular capillary pressure during cooling changed into an excessive pressure after rewarming. The glomerular filtration rate (GFR) decreased by almost 50 % during hypothermia and increased to nearly baseline values after rewarming. The fall in GFR assumingly resulted from a decrease in filtration net driving pressure at unchanged filtration coefficient. Both proximal and distal tubular fluid flow decreased, but fractional reabsorption remained unchanged. Interestingly, urine flow increased by more than 200% during hypothermia, mainly as a result of reduced fluid reabsorption in the distal tubules, and returned to slightly above baseline after rewarming (119). These observations were consistent with findings in dogs cooled to 21°C of 73 and 80% reductions of renal cortical and medullary blood flows, respectively, as reported by Anzai et al. (42).

Based on a meta-analysis of data from patients who underwent thoracic aortic surgery in deep hypothermic circulatory arrest, the authors found no evidence that hypothermia *per se* damage the kidneys (120). Another study showed that therapeutic hypothermia prevented neither the development of acute kidney injury nor the requirement of dialysis (121). Recently, Arnaoutakis et al. showed that patients undergoing elective ascending aortic hemiarch repair, showed no difference in rates of acute kidney injury between one group, operated under moderate HCA and anterograde cerebral perfusion, and another group exposed to deep HCA and retrograde cerebral perfusion (122). On the other hand, after injuries, rhabdomyolysis, characterized by increased serum levels of myoglobin, creatine kinases (CK and CK-MB) and transaminases (ASAT and ALAT), often presents with acute renal injury. Evidence is accumulating that even therapeutic hypothermia may facilitate rhabdomyolysis in injured patients (123, 124). To reduce the risks of rhabdomyolysis, experts recommend increased administration of isotonic fluids from the site of accident, and in case of myoglobinuria, sodium bicarbonate solution should be administered for urine alkalinization (123).

Lactic acidosis and electrolyte and fluid disturbances characterize non-survivors of AH. Elevated serum potassium concentration, traditionally a marker of asphyxia, has been considered a limiting factor of successful resuscitation in victims of HCA (18). Attempts on resuscitating HCA patients with serum potassium exceeding 9 mmol/L were considered futile until Dobson and Burgess successfully resuscitated a girl aged 31 months with a serum potassium concentration of 11.8 mmol/L. She had unattended locked herself out from home (outdoor temperature −22°C) after her father had left for work at 02:30 in the morning. Her mother found her pulseless without respiration at 08:10. Two nurse residents provided CPR, and ambulance personnel intubated her at 09:15. Then, ECG showed bradycardia (< 10 beats/minute) without palpable pulses. She arrived at hospital at 09:38 with a rectal temperature of 14.2°C and was connected to CPB for rewarming at 10:10. After successful resuscitation, her condition complicated with gangrene of left leg, necessitating amputation. When followed up at 3 years of age, she was able to walk with a prosthesis. Her development was otherwise unremarkable (125).

Recently, investigators resuscitated a 7 year old boy after an estimated submersion time of at least 83 min in icy sea water presenting on admission to hospital with a potassium of 11.3 mmol/L and a pH of 6.6 (126). However, despite these rare reports, most authors still agree that high potassium values might identify HCA victims in whom death occurred before cooling (127).

A few years ago, investigators reported median potassium concentrations in survivors of HCA of 5.9 mmol/L against 7.7 mmol/L in non-survivors (14). In a retrospective study of avalanche victims, Locher et al. reported a serum potassium concentration of 6.4 mmol/L in one of the patients, which is the highest ever registered in a survivor of avalanche (128). Arterial lactate concentration also has been suggested a role as prognostic factor of AH with refractory cardiac arrest, but no consensus has been reached concerning its concentration limit with a maintained possibility of surviving.

Generally, AH patients with poor outcomes present with lower pH and higher concentrations of potassium, creatinine, sodium and lactate in parallel with more severe coagulation disorders (127). However, according to Mair and co-workers, moderate and severe hyperkalemia in victims of cardiac arrest after prolonged exposure to cold need not necessarily indicate postmortem autolysis. Consequently, decisions to continue or terminate CPR should not base solely on laboratory parameters (129).

### Hypothermia-Induced Microvascular Changes

Victims of severe AH usually present with increases in hemoglobin, hematocrit and blood viscosity (4, 130). Experimental studies reveal that induction of hypothermia is associated with extravasation of water and proteins independent of whether hypothermia is due to surface cooling or core cooling. Investigators have shown that fluids and proteins shifting from the intravascular to the interstitial compartment might result in edema of most organs, except for the lungs, in which fulminant edema often is seen first after rewarming from HCA (131–133). Therefore, investigators recently wondered if hypothermia-induced increase in pulmonary vascular resistance (PVR) could explain this difference (134). They compared fluid filtration rate in normothermic rat lungs perfused with blood at constant flow with two groups of blood-perfused lungs cooled to 15°C; one group perfused at constant flow and one group perfused at constant pulmonary artery inflow pressure (PPA). Increased fluid filtration rate and fulminant edema appeared in hypothermic lungs perfused at constant flow, but significantly less so in lungs perfused at constant PPA that responded more like normothermic controls perfused at constant flow. The findings were interpreted as the result of a more-fold increase in pulmonary vascular resistance (PVR) in the constant PPA group, which reduced microvascular pressure and fluid filtration rate. A similar mechanism could possibly also provide an early protection against lung edema in humans exposed to AH (134).

The mechanisms responsible for hypothermia-induced fluid shifts are poorly understood. Two theories have been proposed; the first states that the decrease in plasma volume could be explained by trapping of plasma within certain parts of the vasculature (135); the second suggests that a net trans-capillary fluid filtration takes place, thus giving rise to hemoconcentration and decreased circulatory volume (17, 136, 137).

In addition to fluid extravasation, water and electrolytes are lost due to “cold diuresis” resulting from peripheral vasoconstriction with increased central pooling of blood and reduced release of antidiuretic hormone. During rewarming vasodilation may add to the hemodynamic effects of fluid loss (138). However, to prevent further heat loss, fluid replacement should only take place with liquids heated to 38–42°C prior to intravenous administration. In a cold pre-hospital environment, intravenous fluids cool rapidly, and cold fluids can exacerbate hypothermia. Moreover, vasodilatation usually accompanies rewarming. Therefore, warm crystalloid fluids should be administered based on general principles for fluid replacement, such as volume status, plasma glucose, electrolyte concentrations and pH measurements. Health personnel also should bear in mind that resuscitation with large volumes of isotonic saline might aggravate acidosis. Vasopressors should be used with caution to antagonize hypotension (59). Notably, these agents can also provoke arrhythmias and compromise peripheral circulation, which is particularly unfortunate in patients at risk for frostbite (139, 140).

### Interaction Between Blood – and Membrane Surfaces in Cooling and Rewarming

Endothelial glycocalyx is a meshwork of glycoproteins with a thickness of from 20 to several hundred nanometers, which plays an essential role for maintaining cell junction integrity (141). Glycocalyx consists of three building blocks, hyaluronan, heparan sulfate and syndecan and separates endothelial cells from the blood stream. By interacting with plasma proteins and lipids, it constitutes an integral part of blood rheology, hemostasis, and defense against intruders. Hyaluronan, a main component of extracellular matrix, is an anionic non-sulphated glycosaminoglycan of significant importance for cell migration and proliferation. Based on experiments on umbilical vein endothelium, investigators recently suggested that increased shear stress augments the storage of hyaluronan in the glycocalyx (142). Heparan sulfate is a native proteoglycan, attached with HS-bridges to cell surface or extracellular matrix. When activated, heparan sulfate displays heparin-like anticoagulant properties (143). Syndecans have strong associations with the actin cytoskeleton with consequences for regulation of cell adhesion and migration. According to Afratis et al., syndecans interact with cell surface receptors, such as growth factor and integrins leading to activation of signaling pathways that are critical for cellular behavior. Syndecans also play a key role in intracellular calcium regulation and homeostasis (144).

Glycocalyx derangement precedes damage to the endothelium (145). In patients undergoing major surgery employing CPB, investigators found more-fold increments in syndecan−1 and heparan sulfate concentrations indicating that these proteoglycans could serve as markers of glycocalyx shedding (146). Studies of coronary vascular permeability concluded that preventing damage to the glycocalyx should be a prioritized goal for cardioprotection in many clinical conditions, including myocardial ischemia, hypoxia and inflammation (147). The glycocalyx degradation products syndecan-1, heparan sulfate and hyaluronan increased as part of the post-cardiac arrest syndrome. Not surprisingly, syndecan-1 and heparan sulfate levels were higher in non-survivors than in survivors of CA (148).

Coronary artery bypass grafting (CABG) is associated with increased glycocalyx shedding if the surgery is performed with CPB as compared with off-pump (149). However, other investigators failed to show differences in peak syndecan-1 concentrations depending on whether patients underwent CABG on- or off-pump, but found that degradation of glycocalyx was preceded by increased levels of atrial natriuretic peptide (150). Rehm et al. also reported increased syndecan-1 and heparan sulfate degradation products after global ischemia during aortic surgery (146). In children, glycocalyx shedding occurred particularly when CPB, aortic clamping and deep hypothermic CA were combined. In contrast, when performed under beating heart conditions, CPB failed to provoke glycocalyx shedding (151). However, so far, no one study has focused specifically on the influence on endothelial glycocalyx of AH *per se*. Thus, the topic needs further elucidation, both experimentally and clinically.

Tissue Factor (TF) triggers the extrinsic coagulation pathway. During exposure to ECLS, complement activation can stimulate release of TF from monocytes. Additionally, TNF-α and IL-6 trigger generation of soluble TF in endothelial cells (152, 153). Moreover, activation of factor X to Xa provokes cleavage of prothrombin to thrombin and subsequently of fibrinogen to fibrin, the ultimate step in clot formation. Thrombin also promotes inflammation through neutrophil activation, and adherence to endothelial cells mediated by adhesion-molecules E-selectin and P-selectin (154). Activated platelets adhere to fibrin deposited on the endothelial surface and stimulate leukocytes to cytokine secretion and TF-expression (152). Endothelial cells generate platelet activating factor (PAF), which activates neutrophils and their generation of inflammatory cytokines (155). Conventional CPB activates the classical complement pathway, the alternative pathway, and the lectin pathway The lectin pathway activates the adaptive and the innate immune systems. The latter systems defend the body against hostile intruders by direct cell lysis or modulation of leucocytes through opsonisation or generation of pro-inflammatory anaphylatoxins. CPB or ECMO with biocompatible membranes, such as the heparin-coating, seems to reduce the intensity of complement activation *in vivo* (156).

Initiation of ECLS causes generation and release of pro- and anti-inflammatory cytokines. Reactive oxygen species (ROS) and cytokines act in concert with complement to stimulate endothelial cells to secrete pro-inflammatory cytokines that interact with leukocytes through adhesion molecules. Expression of P-selectin dominates the early activation of endothelial cells after complement stimulation. Subsequently, TNF-α and interleukin – 1β (IL-1β), overshadow the process, resulting in neutrophil transmigration and tissue infiltration (152). After initiation of ECLS, a slower monocyte activation follows the peak activation of neutrophils. The latter cells degranulate and release cytotoxic enzymes, like elastases, peroxidases, lysozymes, and ROS, causing widespread tissue damage. These constituents appear at high concentrations in bronco-alveolar lavage during ECMO. Monocytes generate pro-inflammatory cytokines that stimulate the extrinsic coagulation system *via* activation of cytosolic TF (152).

Investigators studying the influence of CPB on inflammatory markers during CABG at BCTs of 32 and 36°C, showed no differences in timing or increments in the levels of IL-6, IL-8, IL-10, cortisol, or CRP, within the first 44 h after the start of operation. Postoperatively, bleeding was less in the normothermic group, but transfusion requirements were the same (157). At variance, Grünenfelder et al. showed higher levels of E-selectin, ICAM, IL-6 and IL-8 after 24 h in CABG-patients treated with CPB at a BCT of 34 °C as compared to 24–26°C (158).

Following HCA, the rewarming technique might also affect the inflammatory reaction. *In vitro*, cooling and rewarming of blood from 21 to 38.5°C showed increased neutrophil elastase activity, in parallel with increments in IL-1β, IL-8 and TNF-α (159). A porcine model of HCA assessing rapid vs. slow ECLS rewarming, showed no difference between groups as regards IL-6, TNF-α and neuron-specific enolase (NSE). Moreover, receptor for advanced glycation end products (RAGE), which is a marker of alveolar epithelial injury, was elevated in the rapidly rewarmed group (160).

In their studies of therapeutic hypothermia, Sipos and co-workers evaluated the impact of different intra-arrest hypothermia levels on the expression of selected cytokines and their prognostic value for 9-day survival in pigs. Interestingly, these investigators found that the systemic inflammatory response syndrome after cardiac arrest is characterized by marked increments in plasma IL-6 and TNF-α levels. As a prognostic marker for 9-Day survival of CA, IL-10 was identified with decreasing mRNA levels (161). Moreover, Meybohm et al. also observed a significant reduction in cerebral cortex inflammatory cytokine mRNA expression after HCA, as compared with animals who underwent CA when normothermic. Thus, mild therapeutic hypothermia resulted in decreased expression of typical cerebral inflammatory mediators after CPR (162).

### Immune Modulation/Suppression–Lessons Learned From Therapeutic Hypothermia

Brain injury may occur both after CA (“ischemia”; I) and after ROSC when brain perfusion is reestablished (“reperfusion injury”; R). According to a review by Tahsili-Fahadan and co-workers, inflammatory processes after ischemia-reperfusion (I/R) injury play an important role in the development of neurological damage. For these conditions, therapeutic hypothermia (TH) has documented beneficial effects (163). After I/R injury, several proinflammatory chemokines, and matrix metalloproteinases (MMPs) aggravate brain injury by increasing permeability of the blood brain barrier, thereby increasing brain edema. In animals, increased levels of IL-10, enhanced expression of tumor growth factor β (TGF-β) and insulin-like growth factor-1 (IGF-1), suppress the activities of Th1 and Th2 lymphocytes after ischemia, thereby inducing neuroprotection. However, the role of this kind of immune suppressive therapy in human HCA is still unknown (163). Mitochondrial permeability transition pore (PTP), which is regulated by the matrix protein cyclophilin D (CypD), plays a key role in the pathophysiology of post-cardiac arrest (CA) syndrome. Recently, Jahandiez et al. demonstrated in rabbits who underwent 15 min of CA followed by 120 min of reperfusion, that therapeutic hypothermia limited post-cardiac arrest (CA) syndrome by preventing mitochondrial permeability transition, mainly through a CypD -dependent mechanism (164).

Cho et al. recently reviewed the evidence after hypoxic-ischemic brain injury of neuroprotective effects of immunomodulation, in addition to cooling. These investigators found encouraging results of stem cell therapy in small animals, suggesting augmented hypothermic neuroprotection. However, still the evidence of these effects are conflicting and the authors recommend rigorous testing in translational animal models (165).

### Resuscitation From HCA With ECLS

In 1967 investigators independently reported successful rewarming from AH of two intoxicated patients by means of veno-arterial CPB (166, 167). Since then, rewarming by ECLS has become an established treatment, especially for those presenting with HCA. Compared with other rewarming modalities, ECLS ensures perfusion and oxygenation in addition to core rewarming. The rate of rewarming is also superior to any other technique (168). Swiss and Austrian physicians operating in close proximity to the Alps have worked out protocols integrating rewarming and trauma management from the site of accident and throughout the whole hospital stay (169–171).

ECLS during HCA activates host defense, including both the complement, coagulation, kinin - kallikrein and the fibrinolytic systems, in addition to leucocytes, platelets and inflammatory cytokines (152, 159, 172–176). The magnitude of these combined responses might affect the extent and degree of multiorgan failure and the outcome after rewarming. Surface contact between the ECLS system, coagulation factors XII, XI and high molecular weight kininogens (HMWK), constituting the contact system, activates production of vasodilator bradykinin *via* the kallikrein-kinin system (177). The activated forms have pro-coagulant and pro-inflammatory effects and can potentially stimulate the release of cytokines and interleukins, like TNF-α and IL-10, and the nitric oxide synthase/nitric oxide (NOS/NO) pathway (152). Upon contact with blood, the ECLS surface activates factor XII (Hageman factor) to XIIa and XIIf. The former triggers the intrinsic coagulation pathway and assists factor XIIf in converting pre-kallikrein to kallikrein and with HMWK to bradykinin. Amplification of factors IX, X and XI, activates the common coagulation pathway.

ECLS can be carried out with conventional cardiopulmonary bypass (CPB), a miniaturized CPB (MCPB) or an ECMO system. The choice of ECLS technique depends on the availability, competence, and clinical judgment. Each method has its advantages and pitfalls (178). ECMO and MCPB share the theoretical advantages of active venous drainage, small priming volumes and minimal hemodilution. Most likely, limited blood-surface-exposure, and possibly, the elimination of a blood-air interface attenuate the inflammatory response to ECLS (152, 179, 180). A review discussing interventions aimed at reducing inflammatory responses to CPB identified eight randomized controlled trials (RCT) comparing CPB with MCPB (181). Six of the RCTs using MCPB showed reduced activation of one or more of the inflammatory markers, IL-8, IL-6, activated complement factor 3 (C3a), the cytolytic component of the complement pathway activation C5b-9, integrin CD11b, TNF-α, neuron-specific enolase (NSE), and thromboxane B2. Additionally, three studies showed reduction of one or more of the clinical endpoints: ICU stay, ventilator time and cardiac troponin I (cTnI) plasma concentration (181).

Of note, in hypothermic piglets, CPB caused greater extravasation as compared to normothermic controls (137). Hyperoncotic priming solutions, like hydroxyethyl starch, and anti-inflammatory drugs, such as methylprednisolone, vitamin C, or α-tri-inositol given as pretreatment, were unable to prevent the increased fluid extravasation rate during hypothermic CPB (182, 183). However, in dogs resuscitated from HCA, addition of the oxidant scavenger N-acetylcysteine (NAC) to the CPB priming solution, reduced the inflammatory response during rewarming. Following exposure to deep hypothermia and ischemia-reperfusion combined, lungs pretreated with NAC demonstrated increased glutathione concentration with less deterioration of lung mechanics and gas exchange (184).

In comparison with CPB, ECMO has the additional advantage of extending cardiopulmonary support for days if the patient displays cardiopulmonary insufficiency after rewarming. In pigs, rewarming with ECMO restored MAP, CO, ḊO_2_, and blood flow to the heart and parts of the brain. Perfusion of the kidneys, stomach, liver, and spleen remained significantly reduced. In comparison, during CPR oxygen delivery (ḊO_2_) and O_2_ uptake (V°O_2_) fell to critically low levels, although, a small increase in lactate and a modest drop in pH indicated the presence of maintained aerobic metabolism (185). A few years ago, Ruttmann and co-workers retrospectively compared 34 patients with HCA rewarmed with CPB and 25 patients rewarmed with ECMO. Multivariate analysis showed a 6.6-fold (95_CI%_ 1.2–49.3) increased chance of surviving with ECMO in comparison with CPB (186). Recently, we confirmed the early finding of Farstad et al. that a woman has a greater chance of surviving HCA as compared to a man. Moreover, that rewarming with ECMO increases the chance of surviving as compared with CPB. However, in a subset of patients from whom we found individual data, serum potassium (*n* = 177) was significantly higher in the CPB group as compared with the ECMO group. By removing 10 patients with s-K^+^ ≥ 11.8 mmol/L from the analysis, differences in survival between ECMO-treated and CPB-treated patients vanished, which is consistent with recent findings of Pasquier et al. (15, 24).

## Summary

- The update surveys physiological changes associated with AH, HCA and rewarming on various organ systems.
- In hypothermic individuals, a decrease in metabolic rate of 6–7 % per °C fall in body temperature, makes it possible to cover the requirement for oxygen even during manually performed CPR over a sizeable period.
- AH hampers the coagulation, the kinin–kallikrein, and the fibrinolytic systems.
- AH inhibits liver enzymes involved in the coagulation cascade, causing reduced prothrombin generation, which gives rise to increased bleeding after injuries.
- During hypothermia, clearance of drugs from the circulation may be hampered, as characterized by reduced elimination of midazolam secondary to declined activity of the P450 enzyme family.
- During hypothermia, renal blood flow and glomerular filtration rate decrease, but urine flow increases, because of reduced fluid reabsorption in the distal tubules.
- In hypothermic subjects, defibrillation and the use of inotropic or vasoactive drugs is not recommended until BCT rises to > 30°C after rewarming.
- After rewarming from AH or HCA, acute myocardial failure might occur, called “rewarming shock.”
- Adjustment of acid-base balance in hypothermic children should follow the “pH-stat strategy”, i.e., correcting pH and blood gases analyzed at 37°C back to the patient's BCT.
- Adjustment of acid-base balance in hypothermic adults with HCA should follow the “alpha-stat strategy,” i.e., employing the values measured at 37°C directly without correcting to the actual BCT.
- The chance of surviving HCA is significantly higher after rewarming with ECMO, as compared to CPB, and in patients with witnessed compared to unwitnessed HCA.
- Male sex, high initial body temperature, low pH, and high s-K+ are factors associated with low surviving chances HCA.
- Avalanche victims have the lowest probability of surviving HCA.
- Rewarming victims of HCA with a serum potassium exceeding 12 mmol/L and a burial time of >30 min after avalanches with no air pocket, most probably be futile.

## Author Contributions

Colleagues at UiT The Arctic University of Norway (TN, ER, TT, and LB), Tromsø, Norway and at the Department of Anesthesiology and Intensive Care, the North-Western State Medical University (KL), St. Petersburg, the Nikiforov Russian Center of Emergency and Radiation Medicine (ES), St. Petersburg and the Northern State Medical University (MK), Arkhangelsk, Russia, collaborated on writing this update on physiological changes in accidental hypothermia and rewarming. LB drafted the manuscript together with TN. ER performed and updated the literature searches, as outlined in Methods. All authors made important contributions to the study, revised the manuscript, read, and approved the final version.

## Funding

The study was supported by grants provided by the Norwegian Center for International Cooperation in Education, Project Number CPRU-2015/10021.

## Conflict of Interest

The authors declare that the research was conducted in the absence of any commercial or financial relationships that could be construed as a potential conflict of interest.

## Publisher's Note

All claims expressed in this article are solely those of the authors and do not necessarily represent those of their affiliated organizations, or those of the publisher, the editors and the reviewers. Any product that may be evaluated in this article, or claim that may be made by its manufacturer, is not guaranteed or endorsed by the publisher.

## Glossary

ADP: Adenosine diphosphate
AH: Accidental hypothermia
ALAT: Alanine aminotransferase
ASAT: Aspartate aminotransferase
ATP: Adenosine triphosphate
ATPase: Group of enzymes catalyzing hydrolysis of ATP to ADP
BAT: Brown adipose tissue
BC: Before Christ
BCT: Body core temperature
°C: Degree(s) Celcius
CA: Cardiac arrest
CAR: Cerebral autoregulation
Ca^2+^: Calcium ion
CABG: Coronary artery bypass grafting
cAMP: Cyclic adenosine monophosphate
CI: Confidence interval
CO: Cardiac output
CO^2^: Carbon dioxide
CPB: Cardiopulmonary bypass
CPP: Cerebral perfusion pressure
CPR: Cardiopulmonary resuscitation
CypD: Matrix protein cyclophilin D
ḊO^2^: Oxygen delivery
ECLS: Extracorporeal life support
ECG: Electrocardiogram
ECMO: Extracorporeal membrane oxygenation
EEG: Electroencephalogram
HCA: Hypothermic cardiac arrest
HMWK: High molecular weight kininogens
hr(s): Hour(s)
ICU: Intensive Care Unit
IGF-1: Insulin-like growth factor-1
IL-6: Interleukin 6
IL-8: Interleukin 8
ISS: Injury Severity Score
MAP: Mean arterial pressure
MMPs: Matrix metalloproteinases
NSE: neuron-specific enolase
OR: Odds ratio
PaO^2^: Arterial partial pressure of oxygen
PaO^2^/FiO^2^: Ratio of arterial oxygen tension to inspired gas oxygen fraction
PEA: Pulseless electrical activity
PEEP: Positive end-expiratory pressure
PET/CT: Positron emission tomography/computed tomography
pH: = −log^10^[H^+^]; literally, the negative logarithm base 10 of the hydrogen ion concentration in a solution
PPCPB: Portable percutaneous cardiopulmonary bypass system
PTP: Mitochondrial permeability transition pore
PVR: Pulmonary vascular resistance
PROSPERO: International Prospective Register of Systematic Reviews
PVR: Pulmonary vascular resistance
RAGE: Receptor for advanced glycation end products
RR: Relative risk ratio
RBF: Renal blood flow
RCT: Randomized controlled trial
ROSC: Return of spontaneous circulation
SD: Standard deviation
s-K^+^: Serum potassium
s-Na: Serum sodium
TGF-β: Tumor growth factor -β
Th-1 and Th-2: Lymphocytes providing immune protection against intra – and extracellular intruders
TRH: Thyrotropin-Releasing Hormone
VF: Ventricular fibrillation
VO^2^: Oxygen uptake
